# Aberrant plasticity of peripheral sensory axons in a painful neuropathy

**DOI:** 10.1038/s41598-017-03390-9

**Published:** 2017-06-13

**Authors:** Takashi Hirai, Yatendra Mulpuri, Yanbing Cheng, Zheng Xia, Wei Li, Supanigar Ruangsri, Igor Spigelman, Ichiro Nishimura

**Affiliations:** 1 Jane and Jerry Weintraub Center for Reconstructive Biotechnology, Division of Advanced Prosthodontics, UCLA School of Dentistry, Los Angeles, CA 90095 USA; 2 Division of Oral Biology and Medicine, UCLA School of Dentistry, Los Angeles, CA 90095 USA; 3 Department of Orthopaedic and Spine Surgery, Tokyo Medical and Dental University, Tokyo, 113-0034 Japan; 4 Division of Biostatistics, Dan L Duncan Cancer Center and Department of Molecular and Cellular Biology, Baylor College of Medicine, Houston, TX 77030 USA; 5 Department of Molecular Microbiology and Immuology, Oregon Health & Science University, Portland, OR 97273 USA; 6 Neuroscience Research and Development Group, Department of Oral Biology, Faculty of Dentistry, Khon Kaen University, Khon Kaen, 40002 Thailand; 7 NeuroEngineering, Department of Bioengineering, UCLA Henry Samueli School of Engineering and Applied Science, Los Angeles, CA 90095 USA

## Abstract

Neuronal cells express considerable plasticity responding to environmental cues, in part, through subcellular mRNA regulation. Here we report on the extensive changes in distribution of mRNAs in the cell body and axon compartments of peripheral sensory neurons and the 3′ untranslated region (3′UTR) landscapes after unilateral sciatic nerve entrapment (SNE) injury in rats. Neuronal cells dissociated from SNE-injured and contralateral L4 and L5 dorsal root ganglia were cultured in a compartmentalized system. Axonal and cell body RNA samples were separately subjected to high throughput RNA sequencing (RNA-Seq). The injured axons exhibited enrichment of mRNAs related to protein synthesis and nerve regeneration. Lengthening of 3′UTRs was more prevalent in the injured axons, including the newly discovered alternative cleavage and polyadenylation of NaV1.8 mRNA. Alternative polyadenylation was largely independent from the relative abundance of axonal mRNAs; but they were highly clustered in functional pathways related to RNA granule formation in the injured axons. These RNA-Seq data analyses indicate that peripheral nerve injury may result in highly selective mRNA enrichment in the affected axons with 3′UTR alterations potentially contributing to the mechanism of neuropathic pain.

## Introduction

Neurons in the central and peripheral nervous systems receive physiological stimuli as well as pathological insults that transiently or permanently modulate neuronal function. Neural plasticity, broadly described as the changes in neuronal morphology^1^, cell-cell interaction^2^, and gene expression^3^, allows neurons to efficiently respond and adapt to those environmental challenges. The uniqueness of neurons is highlighted by their exceptionally complex compartmental diversity. Within a single neuron, various cellular compartments may respond differently to environmental stimuli by initiating localized synaptic- and non-synaptic plasticity.

In recent years, co/post-transcriptional mechanisms such as mRNA modification, axonal transport and subcellular protein synthesis have been recognized as key regulators of spatial-temporal control of neural plasticity^4–6^, including synaptic plasticity and memory^7^. The initiation of translation is rigidly regulated by the formation of eukaryotic initiation factors (eIFs) and ribosomal complex that binds to the 5′-cap structure of mRNA^8^. All necessary factors for protein translation are found in the axon of peripheral neurons^9^. Using hyperalgesic priming models, it has been shown that translational regulation of localized mRNAs in sensory axons plays an important role in the maintenance of chronic pain^10, 11^.

Local protein translation depends on transport of mRNA from the cell body to distal sites. Axonal mRNA transport and localization are mostly controlled by untranslated regions (UTR) at the 3′ ends. With the advent of high throughput RNA sequencing (RNA-Seq), genome-wide analyses of mRNAs revealed a widespread use of alternative cleavage and polyadenylation yielding multiple 3′UTR structures^12^. The 3′UTR contains *cis*-binding sites for micro RNAs (miRNAs) and regulatory RNA binding proteins (RBPs). The alternative selection of canonical polyadenylation sites results in the variable lengths of the 3′UTR, which may generate different recognition *cis*-acting binding sites to miRNAs and/or RBPs, ultimately modulating mRNA stability, its subcellular localization, and protein synthesis^12–15^.

Pathological stimuli or injury to peripheral nervous system can result in painful neuropathies that commonly share clinical features such as light touch-evoked pain (allodynia), burning sensation, exaggerated responses to noxious stimuli (hyperalgesia), and either spontaneous or evoked unpleasant abnormal sensations (dysesthesia)^16^. These symptoms are widely considered due to the hyperexcitability of primary sensory neurons^17–20^ and the ectopic activation of the voltage-gated ion channels including the sensory neuron specific voltage gated sodium channel 1.8 (NaV1.8)^21^. It is highly conceivable that during the development of a painful neuropathy, neuronal cell bodies, axons and synapses undergo maladaptive plasticity as a response to the etiological events^22, 23^. The present study used RNA-Seq analysis of rat dorsal root ganglia (DRG)-derived neuronal cell bodies and axons after sciatic nerve injury to characterize the compartmental distribution and 3′UTR changes of mRNAs.

## Results

### Axonal RNA preparation from rat DRG cells after sciatic nerve entrapment (SNE)

**Figure 1:**
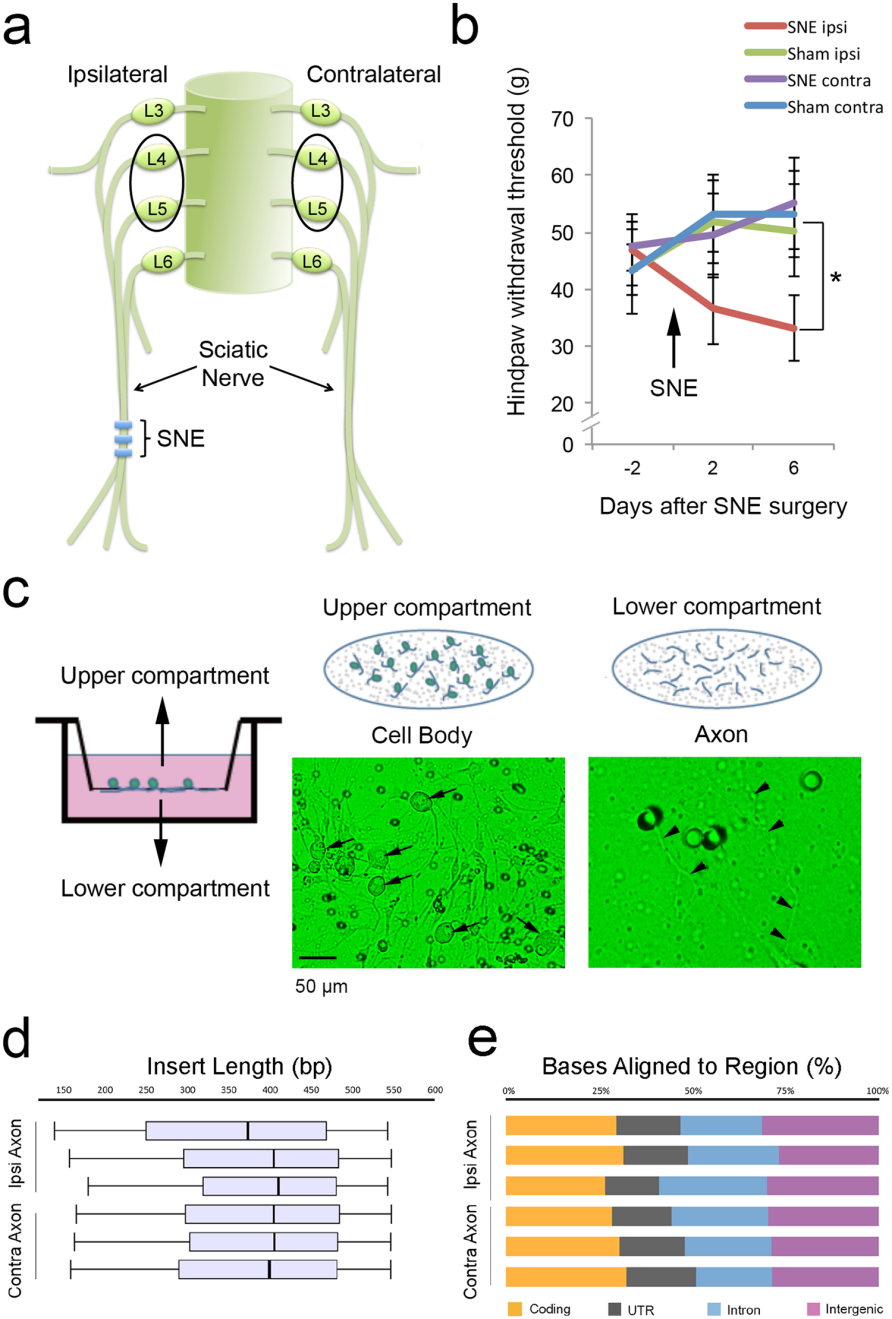
RNA-sequencing of an *in vitro* experimental model involving rat sciatic nerve chronic injury. (**a**) Adult male Sprague-Dawley rats underwent sciatic nerve entrapment (SNE) surgery by placing loosely fit polyethylene cuffs to one of sciatic nerves. (**b**) All rats with SNE or sham surgeries were tested for the development of neuropathic pain symptoms using hindpaw withdrawal threshold to mechanical stimuli (mean ± SEM). (**c**) Once neuropathic pain symptoms developed, L4 and L5 dorsal root ganglia (DRG) were harvested and acutely dissociated. Cells were cultured in the modified Boyden chamber system. While neuronal cell bodies (arrows) remained on the upper compartment, outgrowing neurite processes (arrowheads) were able to pass through pores to the lower compartment. RNA samples were separately prepared from the upper and lower compartments, of which the former represented predominantly cell body RNA and the latter represented purely axonal RNA. RNA-Seq datasets were obtained from 3 independent RNA samples of cell body and axon materials derived from DRG ipsilateral or contralateral to SNE injury. (**d**) The RNA-Seq data were obtained from equivalent size of inserts. (**e**) RNA-Seq reads among different samples similarly distributed to coding as well as multiple forms of non-coding sequences.

Experimental manipulations of rat sciatic nerve by chronic constriction injury (CCI)^24, 25^ and its variant sciatic nerve entrapment (SNE) injury^26–28^ have been shown to induce a painful neuropathy in the ipsilateral hindpaw. The intact DRG and nerve proximal to the site of constriction/entrapment provide a convenient model for molecular biological experiments. In the present study, injury to the rat sciatic nerve was accomplished by unilateral SNE surgery (Fig. 1a). The development of neuropathy symptoms was detected by the decreased withdrawal thresholds to mechanical stimuli applied to hindpaw ipsilateral to SNE surgery, which was significantly different from the measurements of contralateral hindpaw as well as sham operated animals 6 days after surgery (Fig. 1b). Seven days after SNE surgery, lumbar DRG (L4 and L5) were harvested and acutely dissociated. To obtain sensory neuron axonal RNA apart from somatic-derived RNA, the dissociated DRG cells were cultured in the Boyden chamber system (Fig. 1c). In this system, DRG neurons remained on the upper compartment, whereas axon-like neurites passed through the porous membrane and extended to the lower compartment. After 2 days of culture, RNA samples were prepared separately from the upper and lower compartments.

### High throughput RNA-Sequencing (RNA-Seq)

After the quality and quantity of RNA were determined, we prepared 3 independent RNA samples from each cell body and axon group ipsilateral and contralateral to SNE. A total of 12 RNA samples were subjected to RNA-Seq using a commercially available protocol (Hi-Seq, Illumina, San Diego, CA). About 67 to 78% (average 74%) of 16 to 31 million total raw reads (average 24 million raw reads) were uniquely aligned to Rat genome sequence (Build rn5) (Table S1). Sequencing and mapping quality were assessed using RSeQC, which showed that the average mRNA insert fragment size was 399 bp ± 15 bp; SD) (Fig. 1d). The distribution of mapped reads on the genome was consistent among samples (Fig. 1e). These aligned reads were subsequently analyzed by Cufflinks suites for transcript assembly, abundance evaluation, and normalization (Table S2). An average of 67,375 transcripts among samples was obtained. We identified 19,469 (±74; SD) and 19,655 (±62; SD) transcripts with known function in the SNE-conditioned (ipsi) and the control (contra) axons, respectively, with 19,027 common transcripts among the 6 samples. The number of potentially novel isoforms was 5,061 (±384; SD) and 5,647 (±153; SD), and the number of unknown or intergenic isoforms was 40,137 (±17653; SD) and 38,566 (±8393; SD) in ipsi- and contralateral axon groups, respectively.

### Transcriptome analysis for modulated gene expression

**Figure 2:**
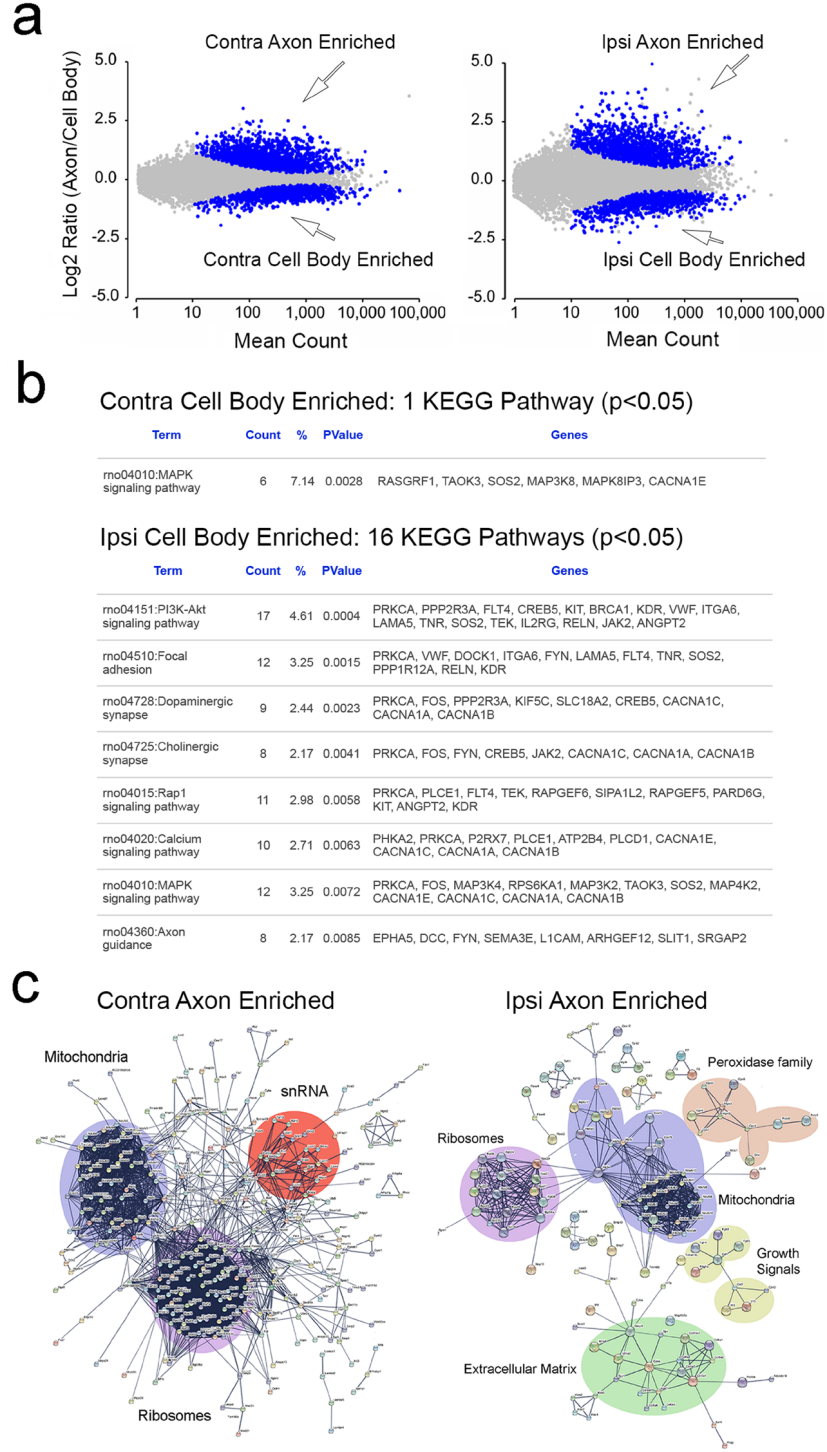
RNA-Seq analysis of enriched mRNAs in cell body and axon compartments. (**a**) Analysis of RNA-Seq reads processed by STAR aligner and DESeq2 revealed mRNA profiles in axon and cell body compartments of SNE ipsi and contralateral DRG neurons. In the log ratio and mean average (MA) plots, blue dots represent mRNAs exhibiting the compartment specific enrichment (q < 0.05). Note that SNE conditioning increased log raitos as shown by MA plot. (**b**) The mRNAs in the cell body compartments with the expression level ≥ 2.0 over the axon compartments were subjected to KEGG analysis. While 1 KEGG pathway was identified in mRNAs enriched in the control (contra) cell body compartment, 16 KEGG pathways were found in SNE-conditioned (ipsi) cell body compartments. (**c**) The enriched mRNAs in the axon compartments were subjected to global protein network analysis using the STRING tool. The contra axon compartment was largely composed of mRNAs associated with protein synthesis (ribosomal structure and catalytic enzymes) and mitochondrial structure and functions, as well as small nuclear RNA associated protein complex. In the ipsi axons, in addition to the ribosomal and mitochondria-associated mRNAs, an mRNA cluster of extracellular matrix (type VIII and type XV collagens) and matrix metalloproteinase was identified. In addition, smaller clusters of peroxidase family and growth signals were detected.

Neural plasticity in response to extracellular stimuli can occur partly through axonal redistribution of specific mRNAs thus altering their steady state levels in the subcellular domains. To determine the relative abundance of specific mRNAs in the axon and cell body compartments, we performed gene expression analysis of the RNA-Seq reads comparing axon to their cell body compartment on the SNE-ipsi and contralateral sides. Our analysis identified 2,146 and 2,716 mRNAs with significantly modulated expression (q < 0.05) between the axon and soma compartments on the SNE-conditioned (ipsi) and control (contra) sides respectively (Fig. 2a). Among those, 387 and 88 unique mRNAs were found enriched (defined to be at least two-fold increase, log2FC > 1) in the SNE-conditioned (ipsi) cell body and the control (contra) cell body compartments over the putative axon compartments, respectively. To consider the activated molecular pathways, we submitted the identified enriched mRNAs of ipsi and contra cell body compartments to ontogeny analysis using DAVID, which identified 16 and 1 Kyoto Encyclopedia of Genes and Genomes (KEGG) pathways, respectively (Fig. 2b). The identified KEGG pathways in the ipsi cell body compartment have been associated with peripheral neuropathy^29–33^ or neuron growth and development^34, 35^.

In ipsi and contralateral axon compartments, 974 mRNAs on the SNE-conditioned (ipsi) side and 578 mRNAs on the control (contra) side showed at least two-fold difference (log2FC > 1), of which 424 mRNAs were common. The enriched mRNAs in the contra axon compartment as compared to the cell body were predominantly related to the structure and function of ribosomes and mitochondria, which generated a tightly connected network including an additional network of small nuclear RNA-related transcripts (Fig. 2c). The enriched mRNAs in the SNE-conditioned (ipsi) axon compartment also formed a network of ribosomes and mitochondria-related genes; however separate gene networks were identified of extracellular matrix-related transcripts (i.e., Col3, Col6, Col8 Col15, Spn, Spp1, Mmp7, Mmp8, Mmp9, Cd44), growth signal receptors (i.e., Fgfr2, Fgfr3, Fgfr4, Pdgfra, Csl1, Cls2, Il14, Il15, Cd40), and peroxidase family (i.e., Gpx4, Gpx5, Gpx6, Gpx7, Gpx8, Prdx5, Prdx6, Mgst2, Mgst3) (Fig. 2c). The list of mRNAs in the SNE-conditioned (ipsi) and the control (contra) axons was further analyzed for ontogeny pathways. As expected, transcripts related to ribosomes and mitochondria were common in ipsi- and contra axons. KEGG analysis of those enriched mRNAs in the SNE-conditioned (ipsi) axon compartment further identified clusters of genes with inter- and intracellular signaling pathway functions as well as autocrine/paracrine systems stimulating growth and differentiation (Fig. S1).

### Dynamic analysis of alternative polyadenylation from RNA-Seq (DaPars)

**Figure 3:**
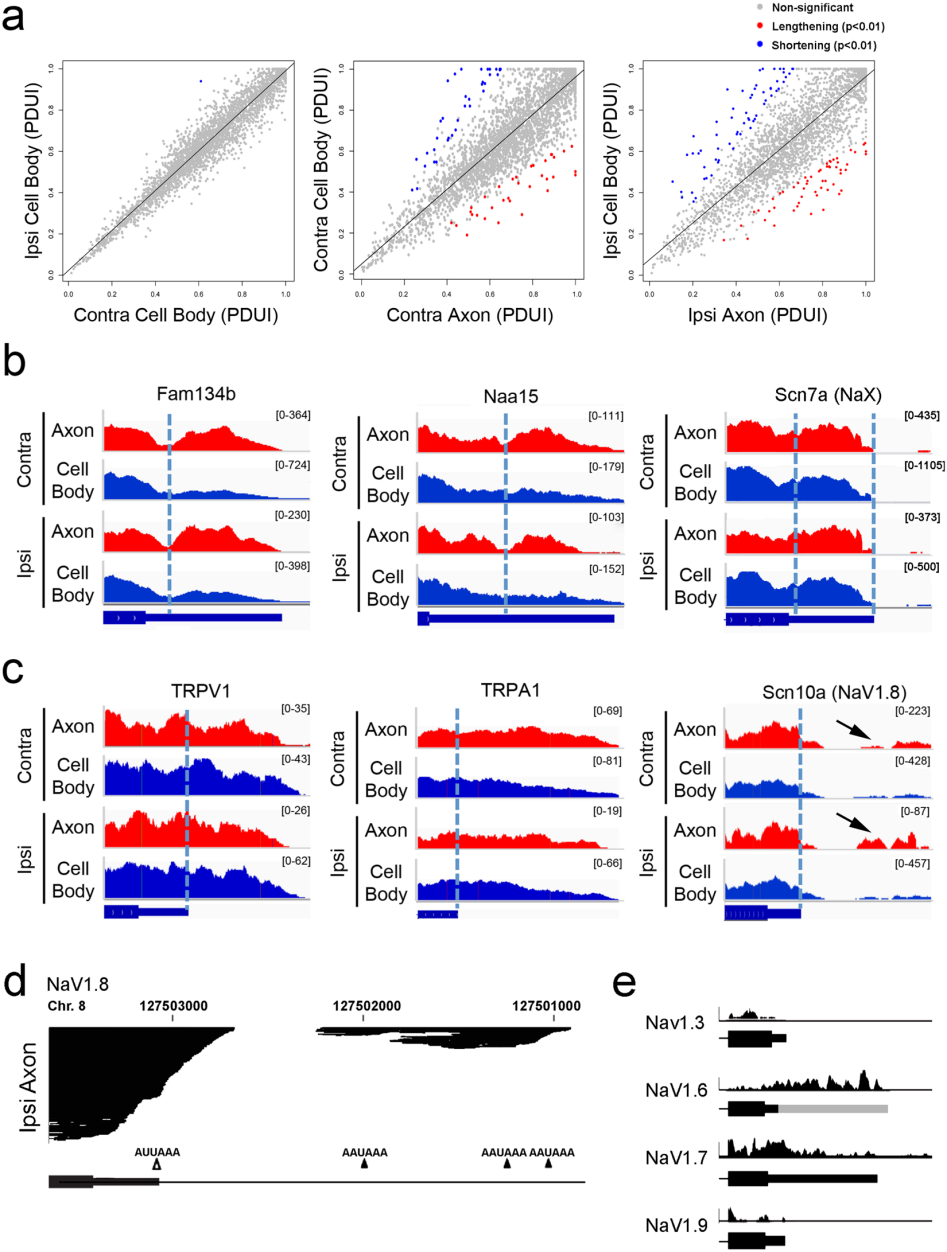
The 3′UTR analysis was carried out by Dynamic analyses of Alternative Polyadenylation from RNA-Seq (DaPars)^51^. (**a**) The Differential Percentage of Distal PolyA site usage index (DPDUI) did not detect significant differences between RNA samples of ipsi and contralateral cell body groups. However, the axon samples showed either lengthening or shortening of 3′UTRs as compared to the corresponding cell body samples at the adjusted significance level of p < 0.01. (**b**) DaPars identified alternatively extended 3′UTRs in ipsi and contra axons of Fam134b, Naa15 and Scn7a (NaX) mRNAs. (**d**) DaPars further predicted “novel” differential 3′UTR structures that were not identified in the rat genome sequence (Build rn5). TRPV1 and TRPA1 exhibited extended 3′UTRs in cell body and axon groups. Scn10a encoding voltage-gated sodium channel 1.8 (NaV1.8) exhibited the possible presence of an alternative exon (arrows) distal to the annotated 3′UTR predominantly in the ipsi axon samples. (**d**) RNA-Seq reads of Scn10 (NaV1.8) in the ipsi axon were found accumulated distal to the annotated 3′UTR. The genomic DNA sequence suggested the presence of multiple polyA signal consensus sequences within the area of accumulated RNA-Seq reads. (**e**) NaV isotypes: NaV1.3, NaV1.7 and NaV1.9 did not show alternative 3′UTR structures, whereas NaV1.6 exhibited the 3′UTR extension in cell body and axon samples. The long 3′UTR has not been reported in rats; however, human and mouse NaV1.6 were reported to have alternative 3′UTR structures (gray bar).

DaPars is a linear regression algorithm aiming to determine known and *de novo* alternative polyadenylation (APA) events at the 3′UTR and differentiate the occurrence of APA using the RNA-Seq read density data. Comparison between the ipsi and contra cell body samples revealed limited use of APA sites that was found only in 16 transcripts even when the significant level was adjusted to p < 0.05. By contrast, mRNAs containing alternative 3′UTR structures were significantly increased in the control (contra) and injury-conditioned (ipsi) axons (Fig. 3a). Based on the DaPars data, we next visualized integrated read fragments at the 3′UTR region on the Broad Integrative Genomics Viewer (Broad IGV). For example, the 3′UTR of Fam134b, Naa15 and Scn7a (NaX) mRNAs showed differential use of “long” 3′UTRs in the axons as compared to the cell bodies (Fig. 3b), whereas TRPV1 and TRPA1 exhibited extended 3′UTR structures in all sample groups (Fig. 3c). The National Center of Biotechnology Information (NCBI) database (www.aceview.org, Rat Sep08, Mouse Jun07 and Human 2010) did not report these extended 3′UTRs of TRPV1 and TRPA1, suggesting these extended 3′UTRs to be novel.

### Identification of new NaV1.8 alternative 3′UTR

The combination of DaPars and the Broad IGV analysis further identified RNA-Seq fragments beyond the end of the annotated 3′UTR in Scn10a (NaV1.8) (Fig. 3c and d). DaPars analysis indicated the presence of extended 3′UTR in Scn10a (NaV1.8) only in the SNE-conditioned (ipsi) axons (p = 0.011). NaV1.8 is one of the voltage-gated sodium channel species found selectively in damage-sensing peripheral neurons and its upregulation was reported in the injured nerves^36, 37^. Other NaV isoforms such as NaV1.3, NaV1.7 and NaV1.9 did not show RNA-Seq fragments beyond the end of the annotated 3′UTR structure; however, NaV1.6 did exhibit RNA-Seq fragments beyond the annotated data of rats (Fig. 3e). Because alternative 3′UTR structures have been reported in human and mouse NaV1.6^38^, it is reasonable to predict the presence of extended 3′UTR in the rat NaV1.6 mRNA.

**Figure 4:**
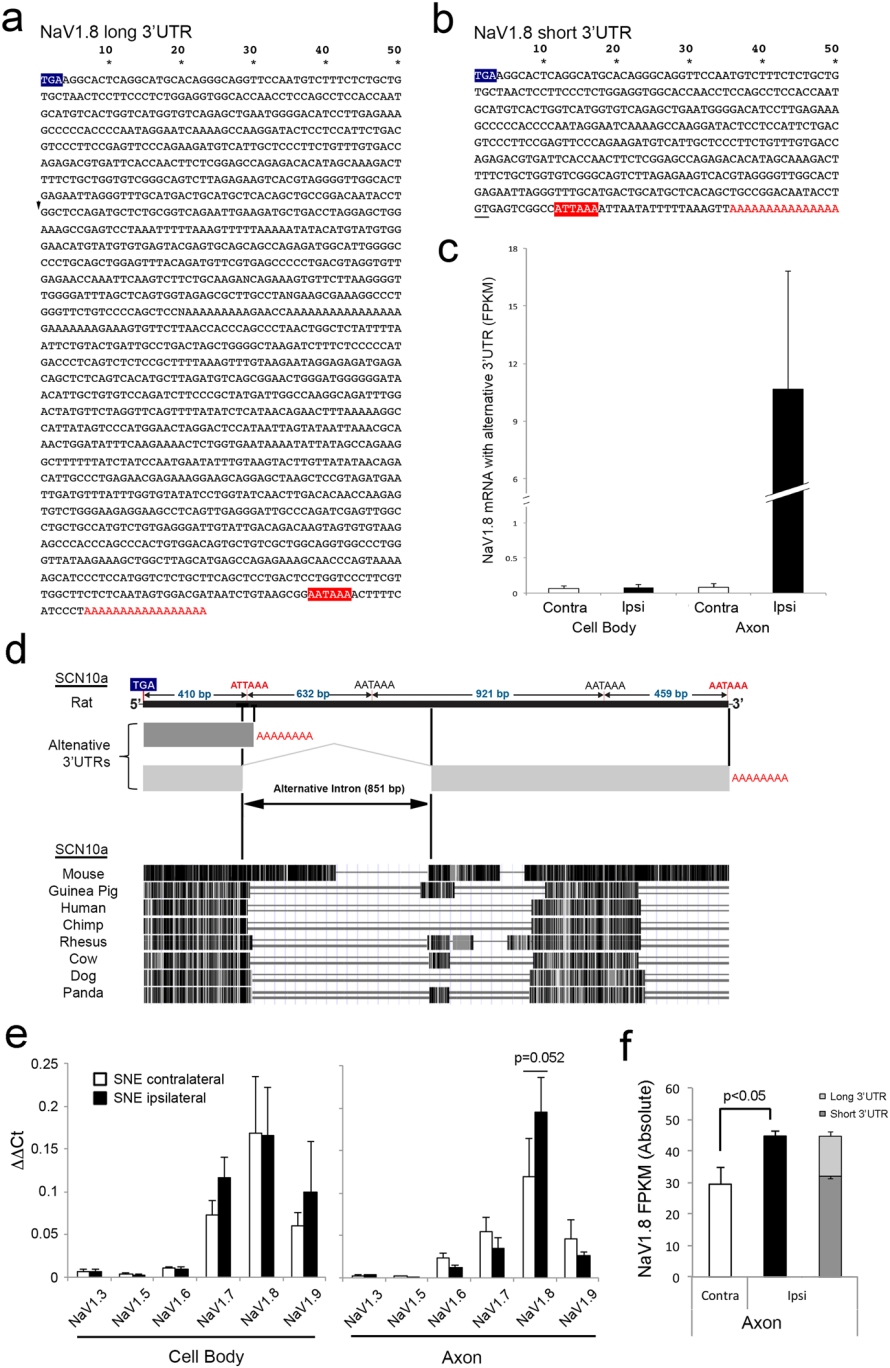
Molecular cloning and sequencing using a combination of targeted PCR and 3′RACE identified the previously unreported long 3′UTR sequence. (**a**) The NaV1.8 long 3′UTR was composed of a 1603 nt-long sequence including the canonical polyA signal (red box) followed by a polyA tail. (**b**) The previously published short 3′UTR sequences of NaV1.8 share the first 400 nucleotides with the newly discovered long 3′UTR and then diverted, as GT in the short 3′UTR (underline in **b**) apparently served as the donor sequence of the alternative intron in the long 3′UTR. (**c**) The use of the distal exon in the long 3′UTR was normalized to the common domain between the long and short 3′UTRs (mean ± SD). The presence of the long 3′UTR appeared to be exclusive in the ipsi axon sample. (**d**) The Scn10a genomic structure revealed that the newly identified exon was separated by an intron (851 bp), alternative splicing of which resulted in the alternative polyadenylation sites. The search of gene sequences of different species suggested significant sequence homology in the domain encoding the short 3′UTR as well as the newly identified alternative exon-intron junction. Furthermore, there was a highly homologous domain in the central part of the new exon encoding the extended part of the long 3′UTR. (**e**) RNA samples used for RNA-Seq were examined for steady state mRNA level changes of NaV isoforms (mean ± SD). The NaV1.8 mRNA level tended to increase in the SNE-conditioned (ipsi) axon, although it did not reach statistical significance (p = 0.052). (**f**) RNA-Seq data were reanalyzed for NaV1.8 FPKM, which revealed that the increase of NaV1.8 mRNA levels in the SNE-conditioned (ipsi) axons was contributed by NaV1.8 mRNA with the long 3′UTR (mean ± SD).

The cluster of RNA-Seq reads distal to the annotated NaV1.8 3′UTR localized on rat chromosome 8: 127,501,055–127,502,247, where 3 additional consensus polyA signals were found (AAUAAA; 1041, 1962 and 2421 bp from the stop codon, respectively) (Fig. 3d). Therefore, although alternative 3′UTR of Scn10a (NaV1.8) has not been reported in the NCBI database, we hypothesized the presence of an alternative long 3′UTR in NaV1.8 mRNA. Reverse-transcription polymerase chain reaction (PCR) using a series of PCR primers targeting the presumptive polyA signal sites followed by 3′ rapid amplification of cDNA ends (RACE) (Fig. S2) identified cDNA clones containing an alternative long 3′UTR sequence of NaV1.8 (Fig. 4a). The newly discovered long 3′UTR of NaV1.8 was composed of the first 400-nucleotide sequence identical to the previously reported short 3′UTR (Fig. 4b). The obtained cDNA sequence was cross-referenced with rat genomic DNA sequence (Fig. S2). The beginning of sequence discrepancy between the long and short 3′UTR was highlighted by GC sequence in the short 3′UTR, likely to be used as the intron donor site, resulting in the alternative cleavage of the proximal polyA signal. There was a canonical polyA signal at the end of the long 3′UTR followed by the polyA sequence. Therefore, the newly discovered long 3′UTR of NaV1.8 was generated by alternative cleavage and polyadenylation.

The exon encoding the long 3′UTR was used to assess the relative expression of NaV1.8 in the RNA-Seq data, which demonstrated that the NaV1.8 mRNA containing the long 3′UTR was exclusively observed in the SNE-conditioned axons (Fig. 4c). The genomic sequence similarity among different species indicated the highly conserved sequence in the short 3′UTR domain, the exon-intron junction of the last exon and the central domain of the long 3′UTR (Fig. 4d).

### 3′UTR landscape of sensory axons

The DaPars data demonstrated that the use of alternative short 3′UTR was common (62.8%) in the control (contra) axons, whereas the use of alternative long 3′UTR increased to 49.0% in the SNE-conditioned (ipsi) axons (Fig. S3a). Alternative use of 3′UTRs appeared to be largely independent from the relative abundance of axonal mRNAs. However, 9.4% and 3.7% of mRNA species with alternative 3′UTRs showed significant up- or downregulation (2×) in the SNE-conditioned (ipsi) and control (contra) axons, respectively, in which mRNA species with extended 3′UTRs appeared to associate with the increased steady state levels in axons (Fig. S3b).

When NaV isoforms were evaluated by real-time PCR, we observed a trend of increased NaV1.8 mRNA in the SNE-conditioned (ipsi) axons compared to the control (contra) axons, while other NaV species in the axon did not differ significantly between the SNE-conditioned (ipsi) and control (contra) axons (Fig. 4e). The RNA-Seq data analysis indicated that there was a significant increase in the NaV1.8 mRNA level in the SNE-conditioned (ipsi) axon (Fig. 4f). When the total FPKM of NaV1.8 was recalculated by subtracting the portion of the newly discovered distal most exon as compared to the stop codon reads, the NaV1.8 with the short 3′UTR in the SNE-conditioned (ipsi) axon appeared to remain at the level of the control (contra) axon. NaV1.8 mRNA with the alternative long 3′UTR appeared to be the primary contributor to the increased NaV1.8 mRNA in the injured axon (Fig. 4f).

### Functional clusters of axonal mRNA with alternative 3′UTR

**Figure 5:**
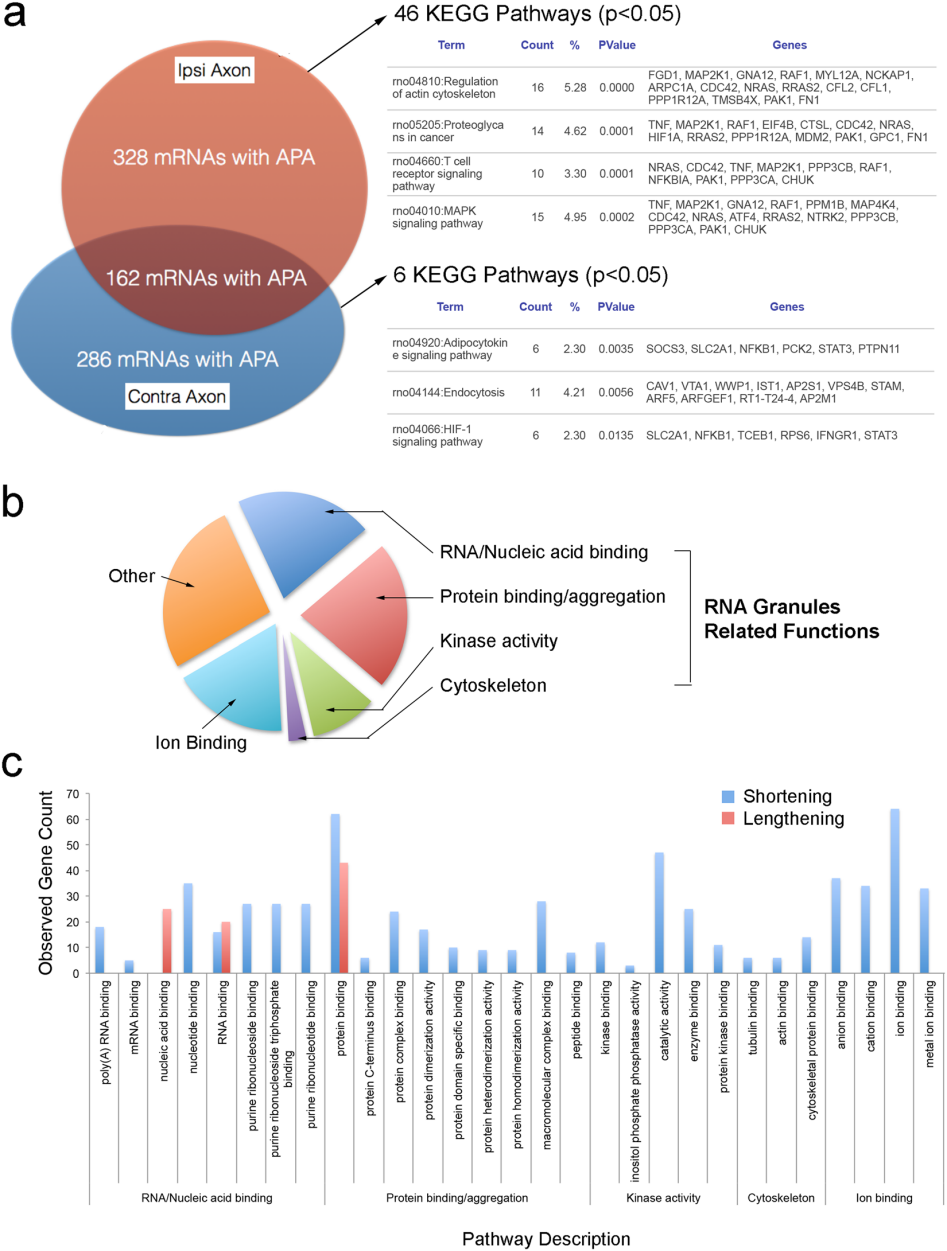
KEGG and Gene ontogeny (GO) pathways of mRNA species containing different 3′UTRs in axons. (**a**) The normalized read coverage in DaPars analysis (p < 0.05) suggested 490 and 448 mRNAs with the use of alternative 3′UTRs in ipsi- and contralateral axons, respectively. Of these mRNAs, 162 species existed in both ipsi- and contralateral axons. Those mRNAs specific to SNE-conditioned and control axons showed strong alliance to 46 and 6 KEGG pathways at the significance level p < 0.05, respectively. (**b**) GO function analysis of mRNAs with alternative 3′UTRs in the SNE-conditioned ipsi axons showed strong clustering in functional pathways, which included RNA binding, protein binding and aggregation, kinase activity, and cytoskeletal molecule binding. These functions appeared to be related to stress granule formation. (**c**) The majority of mRNAs clustered in the stress granule related pathways contained shortened 3′UTRs.

The SNE-conditioned (ipsi) and the control (contra) axon samples contained 494 and 452 mRNAs, respectively, which used different 3′UTRs from the cell body samples (p < 0.05). The axonal mRNAs with differential 3′UTR use shared 162 transcripts; but 328 and 286 transcripts were unique to the ipsi and contra axon samples, respectively. These unique transcripts in the SNE-conditioned (ipsi) axons exhibited strong clustering within a total of 46 KEGG pathways such as the regulation of actin cytoskeleton and MAPK signaling pathway (Fig. 5a). By contrast, the unique transcripts with alternative 3′UTRs in the contra axons clustered in 6 KEGG pathways.

We further analyzed mRNAs with alternative 3′UTRs in the SNE-conditioned (ipsi) axons using Gene Ontology (GO) enrichment analysis for functions. Among 46 functional pathways identified, a large number of transcripts was clustered in pathways related to RNA/nucleic acid binding, protein binding/aggregation and ion binding functions, followed by enzyme/kinase activity and cytoskeleton binding functions (Fig. 5b). We found that an overwhelming number of mRNAs involved in the GO enrichment pathways exhibited shortening of 3′UTRs (Fig. 5c).

## Discussion

Using RNA-Seq strategy, this study demonstrated that the subcellular cell body and axonal compartments underwent robust mRNA modulation after chronic injury to the rat sciatic nerve. The list of mRNAs enriched in ipsi cell body compartment was compared to the microarray data of rat DRG treated with spinal nerve transection^39^, resulting in the match with 61 enriched mRNAs, whereas none matched with the contra cell body compartment. It has been reported that when sciatic nerve was injured prior to acute dissociation and culturing, DRG neurite outgrowth was more robust and peripheral axons appeared to activate their intrinsic growth capacity, as compared to the uninjured DRG culture^40, 41^. The axoplasm of peripheral neurons is characteristically long and axonal mRNA localization is particularly complex and prominent^42^, providing a local regulatory mechanism for responding to acute or chronic injury that may occur at the distant axon^43^. Gumy *et al*. (2011) performed a microarray analysis of axonal RNA isolated from compartmentalized chamber cultures, reporting 812 mRNA species in the axons of naïve and sciatic nerve crush injury rats^44^. Cross-referencing with our data revealed that 44.8% of the commonly upregulated mRNAs in axons and 37.7% of upregulated mRNAs unique to the control (contra) axons were matched. By contrast, only 6.7% of upregulated mRNAs unique to SNE-conditioned (ipsi) axons were matched. After peripheral nerve injury, neurotrophic factors were reported to become available in the surrounding environment, which were thought to promote axonal regeneration^45, 46^. The present RNA-Seq analysis revealed that the increased mRNAs related to growth factor receptors (Fig. 2b and c), if synthesized locally in the injured axons, may provide a capacity for responding to the available neurotrophic factors leading to axonal sprouting and potentially the formation of neuroma. Taken together, these corresponding cross-reference analyses represent a strong validation of our RNA-seq data.

It must be noted that the process of DRG dissociation and plating involves axotomy and loss of supporting satellite cells of all neurons^47^. Consequently, changes in total gene expression in this type of *in vitro* model include changes induced by pre-conditional manipulations such as SNE as well as the axotomy injury. This may have in part contributed to the reduced sensitivity in detection of total gene expression differences between the ipsilateral and contralateral cell bodies. However, our *in vitro* model allowed for mRNA transport between the cell body and axon compartments and appeared capable of detecting SNE-induced changes in the ratios of mRNA species found in the cell body and axon compartments.

Post-translational protein phosphorylation is another powerful regulatory mechanism modulating cellular behaviors through non-genomic activation. Phosphorylation of Erk1/2 MAP kinases in DRG was induced by noxious stimuli^48, 49^ and p-Erk1/2 was found reduced when diabetic rats were treated with gabapentin associated with reduction of thermal hyperalgesia and mechanical allodynia^50^. Any transcriptional assays such as RNA-Seq in the current study are not an appropriate method to address such the post-translational protein modulation. Therefore, we further used our RNA-Seq data for the investigation on the post-transcriptional mRNA modulation.

The most striking observation in this study was the identification of alternative 3′UTR use in axonal mRNAs. The RNA-Seq data were analyzed by a recently developed algorithm; DaPars (dynamic analysis of alternative polyadenylation from RNA-Seq)^51^ to establish 3′UTR landscape in the peripheral nerve cell bodies and axons. There were numerous uses of alternative 3′UTRs in the axon mRNAs (Fig. 3a). The present study further found that the mRNAs with alternative 3′UTRs in the SNE-conditioned axons were strongly clustered within KEGG and GO functional pathways (Fig. 5). mRNAs related to kinases were enriched and/or alternatively polyadenylated in the SNE-conditioned axon. After axonal injury, the mitogen-activated protein kinase (MAPK) was shown to be activated at the lesion site^52^ and is thought to be a nociceptive-induced biochemical signal essential for cross-talk between neurons and glia^53^. MAPK^54^ and phosphoinositide 3-kinase (PI3K)^55–57^ were also shown to facilitate rapid neurite extension *in vitro*. The activation of stress-induced MAPK and PI3K-Akt leads to the phosphorylation of various proteins, including eIF2a^58, 59^. The present study also suggested that those mRNAs with alternative 3′UTRs in the SNE-conditioned (ipsi) axons clustered in the GO functional pathways of RNA binding proteins such as T-cell intracellular antigen-1 (TIA1), Fused in Sarcoma (FUS) and Pumillio homolog 2 (Pum2). These functional clusters are highly relevant to form RNA-protein complexes and cytoplasmic RNA granules^60^ (Fig. 5b,c). Stress granules and processing bodies (P bodies) are both RNA-containing granules that contribute to cellular signaling pathways, metabolic machinery and stress response. Stress granules protect mRNA, stop protein translation, check mRNA fidelity (surveillance and degradation of damaged mRNAs), translocate to other subcellular sites, and re-initiate protein translation^61^. P bodies are enriched in the factors involved in mRNA degradation. Stress granules^42^ and P bodies^62^ have been detected in dendrites and axons of the central nervous system, where they appear to participate in RNA transport and localized protein synthesis. Recently, a functional role of P bodies has also been suggested for peripheral sensory neurons^63^.

The length of mRNA 3′UTR may be globally regulated and neurons were shown to preferentially use distal polyadenylation sites resulting in 3′UTR lengthening^64, 65^. By contrast, mRNAs clustered in the functional pathways related to RNA granules exhibited predominantly shortened 3′UTR (Fig. 5c). The underlining mechanism and functional significance of this phenomenon are beyond the scope of this study. Increases in the expression levels of mRNAs with shortened 3′UTR have been hypothesized to be due to the loss of miRNA and RBP binding^66^. However, this initial hypothesis has been challenged^67^. Recently, the alternative 3′UTR has been postulated to influence the subcellular localization of newly translated proteins and therefore may have an integral biological role beyond modulating mRNA abundance^68^.

Scn10a encoding NaV1.8 showed a cluster of RNA-Seq reads that were located distal to but separated from the annotated 3′UTR. Subsequent cloning and sequencing of cDNA revealed a new exon encoding the long 3′UTR sequence, which was generated through a combination of alternative cleavage and polyadenylation (Fig. 4a and d). Accumulation of NaV1.8 mRNA^37, 69^ and peptide^21, 70^ has been reported in axons after chronic injury. The increased NaV1.8 current and neuronal excitability are thought to contribute to the development of neuropathic pain^71^. Strikingly, NaV1.8 mRNA with the long 3′UTR was present exclusively in the SNE-conditioned axon (Fig. 4c), which may have accounted for the trend towards increased steady state NaV1.8 mRNA level in the ipsi axon (Fig. 4f). The long 3′UTR of NaV1.8 possesses significantly more RBP binding sites (Tables S3 and S4). PTBP1 and Pum2 were among RBPs only found in the extended 3′UTR sequence (Fig. S4), which might play a role in RNA granule formation^72, 73^. It is tempting to speculate that NaV1.8 mRNA with the long 3′UTR might be involved in RNA granule formation in the injured axon.

In conclusion, this study provides new clues to elucidating the molecular mechanisms of neuropathy by RNA-Seq analyses. Our data indicate substantial mRNA modifications localized to the SNE-conditioned axonal compartment and their alignment to highly clustered functional pathways related to nerve regeneration and RNA granule formation may provide a new clue to address the compartment specific molecular pathogenesis in the injured axon. We postulate that aberrant neural plasticity and concordant post-transcriptional regulation of mRNA may be organized, in part, by the generation of ribonucleoparticles within in the axonal compartment, which after local translation, could contribute to neuronal hyperexcitability and the development of injury-induced neuropathy.

## Methods

### Animals

Adult Sprague-Dawley rats weighing 200–300 g were used. All experimental protocols using animals were reviewed and approved by the UCLA Animal Research Committee (ARC# 2006-122) and followed the guideline of PHS Policy for the Humane Care and Use of Laboratory Animals and the UCLA Animal Care and Use Training Manual.

### Sciatic Nerve Entrapment model

Surgical procedure for the SNE model was described previously^74, 75^. Briefly, in anesthetized rats, the left sciatic nerve was surgically exposed, and three polyethylene cuffs (1 mm long, 2.28-mm outer diameter, and 0.76-mm inner diameter) were loosely fitted to the sciatic nerve proximal to the trifurcation of common peroneal, tibial, and sural nerves. Muscle and skin were separately closed. A sham group of rats underwent surgeries without placing the polyethylene cuffs in tandem with the SNE group.

### Behavioral Testing

Measurements of hindpaw withdrawal thresholds to mechanical stimuli were obtained in SNE rats every 2 days as described in detail previously^74, 75^. Hypersensitivity of ipsilateral hindpaws was confirmed for each SNE rat.

### Acutely dissociated DRG cells culture on the Boyden chamber system

Seven days after SNE surgery, L4-L5 DRG were harvested from SNE male Sprague-Dawley rats and acutely dissociated as previously described^74^. Ipsilateral and contralateral DRG neurons from SNE rats were cultured on the Boyden chamber culture system^76^, which separates the upper compartment and the lower compartment by a porous membrane (8 µm pores, BD Falcon, Bedford, MA) coated with Matrigel. Uridine and deoxy 5-fluorouridine were added (final concentration 1.5 mg/mL) into the culture medium to prevent non-neuronal cell proliferation. Cultures were maintained for 48 hrs. Then axons were isolated by carefully scraping the cellular contents from the lower membrane surface with a metal scraper (4 times, alternating direction by 90° each time). The DRG cell bodies on the upper compartment were similarly isolated by scraping the upper surface of the membrane.

Our *in vitro* model allows acutely dissociated DRG neurons to develop long enough neurites, which passed through the membrane pores. In order to increase the amount of RNA collected from the axon compartment, we tested the extended (7-day) culture periods. However, despite the use of uridine and deoxy5-fluorouridine to prevent non-neuronal cell proliferation the 7-day cultures exhibited non-neuronal cell proliferation. This would have contaminated the sought after pure neuronal RNA. It is well known that while adult dissociated DRG neurons retain their phenotypic features shortly after dissociation and plating, the longer they are maintained in culture the less of the original phenotype remains. Willis *et al*. (2005) suggested 18 hours to collect RNA samples from the neurite/axon compartment of the Boyden chamber system^77^. In our study, mature neurite/axon was not consistently organized within this period. Li *et al*. (2004) isolated axons from dissociated DRG cultured for 48 hours and demonstrated axonal presence of eIF-4E^78^. We therefore decided 48 hours as appropriate time to harvest axonal RNA samples in order to obtain sufficient axonal RNA and retaining the phenotypic features of SNE conditioning.

### RNA isolation and quantitative Real Time PCR

Total RNA from DRG cultures was isolated and treated using miRNeasy mini and micro kits (QIAGEN) with DNase I (Ambion, Austin, TX). The steady state mRNA levels of NaV1.3, NaV1.6, NaV1.7, NaV1.8, and NaV1.9 were determined by TaqMan-based real time PCR (RT-PCR): NaV1.3-Rn00565438_m1; NaV1.6- Rn00570506_m1, NaV1.7-Rn00581647_m1; NaV1.8-Rn00568393_m1; and NaV1.9-Rn00570487_m1. The mRNA expression levels were normalized using the comparative CT method.

### RNA-Seq analysis

One hundred (100) ng of total RNA isolated from neuronal cultures was subjected to whole transcriptome amplification using the NuGEN′s Ovation RNA-Seq V2 kit (San Carlos, CA, USA) per manufacturer’s instructions. Amplified cDNA samples were then purified via the MinElute Reaction Cleanup Kit (Qiagen) and sheared into small fragments on the Covaris E210 acoustic focusing instrument (duty cycle: 10%, intensity: 5.0, cycles/burst: 200, duration: 45 seconds, mode: frequency swapping, power: 14 W & temperature: 5.5–6 °C), optimized to achieve an insert size of 350 bp. Paired-end cDNA libraries were prepared with TruSeq Nano DNA Library Preparation Kit (Illumina) according to manufacturer instructions and the final library size distribution was determined using the Agilent Bioanalyzer 2100. Prepared libraries were then sequenced on HiSeq 2500 sequencing system (Illumina) in rapid mode following the cluster generation on cBot. Briefly, cDNA libraries were each diluted to 6 pM and spiked with 1% phiX control to improve base calling while sequencing. A 6 pM dilution of phiX control sample was also prepared for analysis. Following the Illumina cBot and HiSeq protocols, the cDNA libraries and the phiX control underwent cluster generation on a HiSeq PE flow cell v3 followed by sequencing on HiSeq 2500 (Illumina). A paired-end (2 × 101) run was performed using the TruSeq SBS Kit (Illumina). Real-time analysis and base calling were performed using the HiSeq Control Software Version 1.4.5 (Illumina). The resulting base calling (.bcl) files were converted to FASTQ files using Illumina’s CASAVA 1.8 software. The number of reads for each sample type was analyzed using the Student′s t-test in SigmaPlot version 11.0 (Systat Software Inc., San Jose, CA). A p-value < 0.05 was considered significant.

### Mapping of RNA-Seq reads and transcript assembly and abundance estimation using Tophat and Cufflinks

Paired-end fastq sequence reads for each sample were aligned to the UCSC *Rattus norvegicus* reference genome rn5 using TopHat v1.3.0^24^ integrated with Bowtie v0.12.7^25^on cloud computing, BaseSpace (Illumina, CA, USA). The resulting aligned reads were analyzed further by Cufflinks v1.0.3^26^. The aligned reads were assembled into transcripts, either with or without a reference genome, and the expression of those transcripts were reported in Fragments Per Kilobase of exon per Million fragments mapped (FPKM). Cuffdiff analysis was performed, with use of the reference genome, to determine differential expression of known isoforms between ipsi- and contralateral samples.

### Differential gene expression assay

Differential gene expression analysis was performed using the RNA Express application on Basespace, Illumina. Briefly, paired-end reads from all samples were mapped to a rat reference genome (Rn5) using the STAR ultrafast read aligner. Post-alignment and read counting, raw read counts were used for differential gene expression analysis using R and DESeq2. A false discovery rate (FDR) adjusted q value < 0.05 was used to test for significance.

### Dynamic analysis of alternative polyadenylation from RNA-Seq (DaPars)

DaPars algorithm has been previously published^51^. Briefly, DaPars identifies distal polyA sites based on RNA-Seq data. It uses a regression model to infer the exact location of the proximal APA site after correcting the potential RNA-Seq non-uniformity bias along gene body and thus detects statistically significant dynamic APAs. Given two or more RNA-Seq samples, distal polyA site refers to the end point of the longest 3′UTR among all the samples, which is used to identify the proximal polyA within this longest 3′UTR region. To identify possible distal polyA site that might locate outside of gene annotation, DaPars extends the annotated gene 3′end by up to 10 kb before reaching a neighboring gene. RNA-Seq data from all input samples were merged to have a combined coverage along the extended gene model. Differential percentage of distal APA usage index (DPDUI) was determined to detect the most significant APA events.

### The DaPars analysis data that support the findings of this study are available from the corresponding author upon reasonable request

#### Kyoto Encyclopedia of Genes and Genomes (KEGG) analysis and Gene Ontogeny (GO) Enrichment Analysis

Differentially expressed genes and genes with alternative 3′UTRs were subjected to KEGG and GO Enrichment analyses using the online tools. These gene lists were submitted to DAVID^79^ (http://david.ncifcrf.gov) and the String Protein-Protein Interaction Network^80^ (http://string-db.org).

### The KEGG and GO analysis data that support the findings of this study are available from the corresponding author upon reasonable request

#### Visualization of mapped reads

Aligned reads were visualized using the Integrative Genomics Viewer (www.broadinstitute.org/igv/). The output files generated from TopHat were converted into files viewable in IGV on the BaseSpace and then processed further by the “count” function in IGV tools (included with the IGV software) to create an average alignment track viewable as a bar chart. The log2 of the frequency of the reads was plotted to better visualize the extensive range of the read coverage. Individual gene views were created by first merging the TopHat output files from the ipsi- and contralateral samples. These merged files were processed in the same way as above with the “count” function in IGV tools. The raw frequency of the reads was visualized in this case.

#### Identification of RNA-binding protein sites

Position weight matrices (PWMs) for 72 RNA-binding proteins (all species) were bulk downloaded from the RNA-binding protein databank^81^. The match PWM function in v. 2.24.1 of the Biostrings Bioconductor package was used to predict binding sites in the NaV1.8 3′UTR.

#### cDNA cloning, 3′ rapid amplification of cDNA ends (3′RACE) and sequencing

RNA pooled from samples collected from the modified Boyden culture was used for cDNA cloning by reverse transcriptase PCR using 3′UTR targeted PCR primers (Fig. S1) following the established protocol (Platinum Taq DNA polymerase, Invitrogen, Carlsbad, CA). To determine the 3′ end of NaV1.8 transcript, 3′RACE was performed (3′RACE System for Rapid Amplification of cDNA kit, Invitrogen). The cDNA template pool generated by reverse transcription primed with anchored (or “lock-docking”) oligo(dT) primer with an adaptor sequence (5′-GGCCACGCGTCGACTAGTACTTTTTTTTTTTTTTTTT-3′) was then inputted into PCR with specific forward primers designed 368 bp upstream of the annotated polyA site with the adaptor sequence used as the reverse primer (5′-CUACUACUACUAGGCCACGCGTCGACTAGTAC-3′). PCR reactions were then separated on 1.2% agarose gels. All observed PCR products regardless of size were isolated and cloned. The PCR products were sequenced using T3 and T7 primers. Obtained sequences were then aligned to the rat genome (version rn5) using the BLAT feature of the UCSC genome browser.

### Statistical Analyses

Student’s *t* test for two-group comparison and one- or two-way analysis of variance (ANOVA) with Tukey’s multiple comparisons for multi-group comparison were used to analyze RT-PCR and RNA-Seq data with *p* < 0.05 accepted as statistically significant. Repeated measures ANOVA analysis was used to compare ipsi- and contralateral hindpaw sensitivity to mechanical stimuli of SNE injured rats.

## Electronic supplementary material

Supplemental Information
