# Supplementary material for: Aberrant plasticity of peripheral sensory axons in a painful neuropathy
Source: Sci Rep. 2017 Jun 13;7:3407. doi: 10.1038/s41598-017-03390-9 (PMC5469767; doi:10.1038/s41598-017-03390-9)

Aberrant plasticity of peripheral sensory axons in a painful neuropathy
Takashi Hirai, Yatendra Mulpuri, Yanbing Cheng, Zheng Xia, Wei Li, Supanigar Ruangsri, Igor Spigelman and Ichiro Nishimura

Supplementary Information:

Table S1. Summary of *TopHat* Alignment

	**Sample name**	**Number of Reads**	**% Total Aligned**
Ipsilateral Axon	Ipsi-#1	28,765,292	73.32%
	Ipsi- #2	21,230,261	67.62%
	Ipsi-#3	16,439,936	77.09%
Contralateral Axon	Contra-#1	26,041,780	78.24%
	Contra-#2	31,178,011	69.81%
	Contra-#3	26,788,744	76.20%

Table S2. Summary of the number of transcript identified on the *Cufflinks*

	**Sample name**	**Transcript count**	**Transcript Known function**	**Potentially novel**	**Unknown or intergenic**
Ipsilateral Axon	Ipsi-#1	89,192	19,402	5,490	60,509
	Ipsi- #2	56,560	19,456	4,945	29,362
	Ipsi-#3	68,568	19,549	4,748	30,540
Contralateral Axon	Contra-#1	57,682	19,727	5,556	29,492
	Contra-#2	74,635	19,613	5,562	46,051
	Contra-#3	57,617	19,626	5,824	40,156

Aberrant plasticity of peripheral sensory axons in a painful neuropathy

Takashi Hirai, Yatendra Mulpuri, Yanbing Cheng, Zheng Xia, Wei Li, Supanigar Ruangsri, Igor Spigelman and Ichiro Nishimura

Table S3. Predicted cis-sequences for RNA binding proteins of the short 3'UTR

RBP	No. of binding sites	Matching sequence	Matrix ID
MBNL1	5	UGCU	669_20071745
a2bp1	4	GCAUG	36_12574126
RBMX	4	CCAG	922_19282290
A2BP1	3	UGCAUG	37_16537540
FUS	3	GGUG	637_11098054
YTHDC1	2	GCAUGC	969_20167602
EIF4B	2	GGAC	352_8846295
RBMX	2	CCAU	922_19282290
RBMX	2	CCAC	922_19282290
SFRS1	2	AGGA	1173_19561594
ELAVL1	2	GUUU	1170_19561594
HNRNPA1	1	UAGGGU	23_7510636
EIF4B	1	GCCGGAC	351_8846295
sap-49	1	UUGUGA	145_9163526
EIF4B	1	GGAA	352_8846295
KHSRP	1	GUCC	1186_17893325
Vts1	1	GCUGGUG	1176_19561594
YTHDC1	1	GACUGC	969_20167602
SFRS13A	1	AAAGACU	1169_19561594
KHDRBS3	1	AUUAAU	1174_19561594
SFRS13A	1	AGAGAAG	1169_19561594
SFRS1	1	UGGA	1173_19561594
KHDRBS3	1	AUUAAA	1174_19561594
KHDRBS3	1	UUUAAA	1174_19561594
ELAVL1	1	AUUU	1170_19561594

Aberrant plasticity of peripheral sensory axons in a painful neuropathy
Takashi Hirai, Yatendra Mulpuri, Yanbing Cheng, Zheng Xia, Wei Li, Supanigar Ruangsri, Igor Spigelman and Ichiro Nishimura

Table S4. Predicted cis-sequences for RNA binding proteins of the long 3'UTR

RBP	No. of binding sites	Matching sequence	Matrix ID
RBMX	16	CCAG	922_19282290
PABPC1	15	AAAAA	24_7908267
MBNL1	13	UGCU	669_20071745
RBMX	12	CCAU	922_19282290
RBMX	9	CCAC	922_19282290
EIF4B	8	GGAA	352_8846295
KHSRP	8	GUCC	1186_17893325
ELAVL1	8	GUUU	1170_19561594
SFRS1	8	UGGA	1173_19561594
Pum2	7	UGUA	329_11780640
ELAVL1	7	AUUU	1170_19561594
FUS	6	GGUG	637_11098054
SFRS1	6	AGGA	1173_19561594
a2bp1	5	GCAUG	36_12574126
KHDRBS3	5	UUUAAA	1174_19561594
EIF4B	4	GGAC	352_8846295
RBMX	4	CCCG	922_19282290
A2BP1	3	UGCAUG	37_16537540
YTHDC1	3	GAGUAC	969_20167602
sap-49	2	UUGUGA	145_9163526
SFRS9	2	AGGAG	797_17548433
YTHDC1	2	GCAUGC	969_20167602
YTHDC1	2	UCAUGC	969_20167602
RBM4	2	CGCG	1172_19561594
KHDRBS3	2	UAUAAU	1174_19561594
KHDRBS3	2	AAUAAA	1174_19561594
KHDRBS3	2	AUUAAA	1174_19561594
KHSRP	1	CCCCCCCC	1185_17893325
ZFP36	1	AAAAAAAAAG	221_12324455
QKI	1	UACUAAC	149_16041388
SFRS2	1	AGGAGAGAU	953_7543047

Aberrant plasticity of peripheral sensory axons in a painful neuropathy
Takashi Hirai, Yatendra Mulpuri, Yanbing Cheng, Zheng Xia, Wei Li, Supanigar Ruangsri, Igor Spigelman and Ichiro Nishimura

HNRNPA1	1	UAGGGU	23_7510636
NONO	1	AGGGA	488_9001221
EIF4B	1	GCGGAAC	351_8846295
sap-49	1	GUGUGA	145_9163526
EIF4B	1	GCCGGAC	351_8846295
EIF4B	1	GCUGGAA	350_8846295
ybx2-a	1	AAGAUC	114_7499328
PABPC1	1	ACUAACA	950_7908267
EIF4B	1	GCUGGAA	351_8846295
Psi	1	ACUACUUUCC	915_11565747
Psi	1	ACAGCUUGCUC	915_11565747
PABPC1	1	ACAAACC	950_7908267
SFRS9	1	AGGAC	797_17548433
PTBP1	1	AGAUCUUUCUCCCCC	1171_19561594
QKI	1	UACUAACAUG	1215_16041388
YTHDC1	1	GAAUGC	969_20167602
MBNL1	1	CGCU	669_20071745
YBX1	1	UCUGCG	1177_19561594
SFRS9	1	AGCAC	797_17548433
Vts1	1	GCUGGUC	1176_19561594
Vts1	1	GCUGGUG	1176_19561594
YTHDC1	1	GCGUGC	969_20167602
QKI	1	UCUUAACUCC	1215_16041388
YTHDC1	1	GACUGC	969_20167602
Vts1	1	GCUGGAG	1176_19561594
Vts1	1	GCUGGGG	1176_19561594
SFRS13A	1	AAAGAAC	1169_19561594
YTHDC1	1	GAGUCC	969_20167602
SFRS13A	1	AAAGGCC	1169_19561594
SFRS13A	1	AAAGACU	1169_19561594
SFRS13A	1	AGAGAGC	1169_19561594
SFRS13A	1	AAAGAGA	1169_19561594
KHDRBS3	1	AUUAAU	1174_19561594
KHDRBS3	1	UUUAAU	1174_19561594

Aberrant plasticity of peripheral sensory axons in a painful neuropathy

Takashi Hirai, Yatendra Mulpuri, Yanbing Cheng, Zheng Xia, Wei Li, Supanigar Ruangsri, Igor Spigelman and Ichiro Nishimura

SFRS13A	1	AGAGAAG	1169_19561594
KHDRBS3	1	GUUAAU	1174_19561594
KHDRBS3	1	CCUAAA	1174_19561594
SFRS13A	1	CAAGACC	1169_19561594
KHDRBS3	1	CUUAAA	1174_19561594
KHDRBS3	1	CAUAAC	1174_19561594
KHDRBS3	1	ACUAAC	1174_19561594
KHDRBS3	1	GAUAAC	1174_19561594
KHDRBS3	1	CCUAAC	1174_19561594
KHDRBS3	1	CUUAAC	1174_19561594
KHDRBS3	1	UUUAAC	1174_19561594

Aberrant plasticity of peripheral sensory axons in a painful neuropathy
Takashi Hirai, Yatendra Mulpuri, Yanbing Cheng, Zheng Xia, Wei Li, Supanigar Ruangsri, Igor Spigelman and Ichiro Nishimura

Fig. S1: Ninety differentially expressed genes in the ipsi axons as compared to the contra axons were submitted to the Kyoto Encyclopedia of Genes and Genomes (KEGG) analysis. The upregulated (red) and the downregulated (blue) genes were categorized in 7 pathways related to growth signals and their receptors.

#pathway ID	pathway description	observed gene count	false discovery rate	matching proteins
4060	Cytokine-cytokine receptor interaction	7	0.00422	Bmp7, Csf1, Cxcl16, Cxcr4, Egfr, Il11, Il1r1
4151	PI3K-Akt signaling pathway	8	0.00488	Csf1, Egfr, Fgfr2, Igf1, Irs1, Itga11, Sgk1, Spp1
4068	FoxO signaling pathway	5	0.01300	Ccnb2, Egfr, Igf1, Irs1, Sgk1
4640	Hematopoietic cell lineage	4	0.01330	Cd38, Csf1, Il11, Il1r1
5200	Pathways in cancer	7	0.01330	Egfr, Fgfr2, Igf1, Mmp2, Ptgs2, Wnt16, Wnt5a
4960	Aldosterone-regulated sodium reabsorption	3	0.02230	Igf1, Irs1, Sgk1
4510	Focal adhesion	5	0.04400	Egfr, Igf1, Itga11, Myl9, Spp1
5205	Proteoglycans in cancer	5	0.04960	Egfr, Igf1, Mmp2, Wnt16, Wnt5a

Aberrant plasticity of peripheral sensory axons in a painful neuropathy

Takashi Hirai, Yatendra Mulpuri, Yanbing Cheng, Zheng Xia, Wei Li, Supanigar Ruangsri, Igor Spigelman and Ichiro Nishimura

Fig. S2: Rat Scn10a Genomic Sequence (Exon29–Exon30): TGA (blue highlight): termination codon; Turquois highlight with arrow: Reverse transcriptase PCR primers; GT (Dark pink highlight): Alternative cleavage site; Red highlight: canonical polyA signal; Green highlight with arrow; Primer used in 3'RACE.

10 20 30 40 50 60 70 80

* * * * * * * *

GTCTGCTGTTTTCTGCAATCCTTAAGTCACTGGAAAACTACTTCTCCCCGACGCTCTTCCGAGTCATCCGTCTGGCCAGG

ATCGGCCGCATCCTCAGGCTGATCCGAGCAGCCAAGGGGATTCGCACGCTGCTCTTCGCCCTCATGATGTCCCTGCCCGC

CCTCTTCAACATCGGCCTCCTCCTCTTCCTCGTCATGTTCATCTACTCCATCTTCGGCATGGCCAGCTTCGCTAACGTCG

TGGACGAGGCCGGCATCGACGACATGTTCAACTTCAAGACCTTTGGCAACAGCATGCTGTGCCTGTTCCAGATCACCACC

TCGGCCGGCTGGGACGGCCTCCTCAGCCCCATCCTCAACACGGGGCCTCCCTACTGCGACCCCAACCTGCCCAACAGCAA

CGGCTCCCGGGGGAACTGCGGGAGCCCGGCGGTGGGCATCATCTTCTTCACCACCTACATCATCATCTCTTTCCTCATCG

TGGTCAACATGTACATCGCAGTGATTCTGGAGAACTTCAACGTGGCCACCGAGGAGAGCACGGAGCCCCTGAGCGAGGAC

GACTTCGACATGTTCTATGAGACCTGGGAGAAGTTCGACCCGGAGGCCACCCAGTTCATTGCCTTTTCTGCCCTCTCAGA

CTTCGCGGACACGCTCTCCGGCCCTCTTAGAATCCCCAAACCCAACCAGAATATATTAATCCAGATGGACCTGCCGTTGG

TCCCCGGGGATAAGATCCACTGTCTGGACATCCTTTTTGCCTTCACAAAGAACGTCTTGGGAGAATCCGGGGAGTTGGAC **Exon29**

TCCCTGAAGACCAATATGGAAGAGAAGTTTATGGCGACCAATCTCTCCAAAGCATCCTATGAACCAATAGCCACCACCCT

CCGGTGGAAGCAGGAAGACCTCTCAGCCACAGTCATTCAAAAGGCCTACCGGAGCTACATGCTGCACCGCTCCTTGACAC

TCTCCAACACCCTGCATGTGCCCAGGGCTGAGGAGGATGGCGTGTCACTTCCCGGGGAAGGCTATGTTACATTCATGGCA

AACAGTGGACTCCCGGACAAATCAGAAACTGCCTCTGCTACGTCTTTCCCGCCATCCTATGACAGTGTCACCAGGGGTCT

GAGTGACCGGGCCAACATTAACCCATCTAGCTCAATGCAAAATGAAGATGAGGTCGCTGCTAAGGAAGGAAACAGCCCTG

GACCTCAGTGAAGGCACTCAGGCATGCACAGGGCAGGTTCCAATGTCTTTCTCTGCTGTGCTAACTCCTTCCCTCTGGAG

GTGGCACCAACCTCCAGCCTCCACCAATGATGTCACTGGTCATGGTGTCAGAACTGAATGGGGACATCCTTGAGAAAGCC

CCCACCCCAATAGGAATCAAAAGCCAAGGATACTCCTCCATTCTGACGTCCCTTCCGAGTTCCCAGAAGATGTCATTGCT

CCCTTCTGTTTGTGACCAGAGACGTGATTCACCAACTTCTCGGAGCCAGAGACACATAGCAAAGACTTTTCTGCTGGTGT

CGGGCAGTCTTAGAGAAGTCACGTAGGGGTTGGCACTGAGAATTAGGGTTTGCATGACTGCATGCTCACAGCTGCCGGAC

AATACCTGTGAGTCGGCCATTAAAATTAATATTTTTAAAGTTatagcggaagtaacatatcttctgtctgtgtgtgtgcg

cacacgcgtgtgtgtgttgtgagtacacatgtacgcgtgcacttggaggccagaggttaatctctggtgtcattcctcag

gcacttcttgagatgaggtctttcacaggcctggagctctccaaataggtcatgccatgacagggcatgagccccaggga

tctacctacctctgccctccccggctgcaagattataggtacactctccgaatgcctggcacttttacataggttatggg

aggtcaaattcaattcctcatgcttgtttacaaaccaagatactcccagacccctagtgttatatcttaaatatcatttt

atgtgcccccccgttggcattctaagctctgccccaccccacagcttgctctcttaccctctcaccctcttcctcctctg

tcccctctgtacccgttcccctccccctctctccatgtgctcatggctgacctttactcctcttcttctctactcctctt

Aberrant plasticity of peripheral sensory axons in a painful neuropathy

Takashi Hirai, Yatendra Mulpuri, Yanbing Cheng, Zheng Xia, Wei Li, Supanigar Ruangsri, Igor Spigelman and Ichiro Nishimura

cttctccctctgcctccccactccccttttctccctttctccccccccttttctcccctaccaccctcttaactcccgtcc

ccatgccctgaataaactctattctatactataccgtggtgtggctggtccctcaggggaaagagatgccccatcatggg

tctgctgaagcatctcatcccccacacctcagcacacctccatagaacatatccccccttctctttatctttttatagaca

ccacccttacatgtatgatactaacatgtgccatgaactacttttccttcactctcagGGCTCCAGATGCTCTGCGGTCA

GAACTTGAAGATGCTGACCTAGGAGCTGGAAAGCCGAGTCCTAAATTTTTAAAGTTTTTAAAAATATACATGTATGTGGG

AACATGTATGTGTGAGTACGAGTGCAGCAGCCAGAGATGGCATTGGGGCCCCTGCAGCTGGAGTTTACAGATGTTCGTGA

GCCCCCTGACGTAGGTGTTGAGAACCAAATTCAAGTCTTCTGCAAGACCAGAAAGTGTTCTTAAGGGGTTGGGGATTTAG

CTCAGTGGTAGAGAGCTTGCCTAGGAAGCGCAAGGCCCTGGGTTCTGTCCCCAGCTCCGAAAAAAAAAGAACCAAAAAAA

AAAAAGTGTTTTTAACCACCCAGCCCTAACTGGCTCTATTTTAATTCTGTACTGATTGCCTGACTAGCTGGGGCTAAGAT

CTTTCTCCCCCATGACCCTCAGTCTCTCCGCTTTTAAAGTTTGTAAGAATAGGAGAGATGAGACAGCTCTCAGTCACATG

CTTAGATGTCAGCGGAACTGGGATGGGGGGATAACATTGCTGTGTCCAGATCTTCCTGCTATGATTGGCCAAGGCAGATT **Exon30**

TGGACTATGTTCTAGGTTCAGTTTTATATCTCATAACAGAACTTTAAAAAGGCCATTATAGTCCCATGGAACTAGGACTC

CATAATTAGTATAATTAAACGCAAACTGGATATTTCAAGAAAACTCTGGTGAATAAAATATTATAGCCAGAAGGCTTTTT

TATCTATCCAATGAATATTTGTAAGTACTTGTTATATAACAGACATTGCCCTGAGAACGAGAAAGGAAGCAGGAGCTAAG

CTCCGTAGATGAATTGATGTTTATTTGGTGTATATCCTGGTATCAACTTGACACAACCAAGAGTGTCTGGGAAGAGGAAG

CCTCAGTTGAGGGATTGCCCAGATCGAGTTGGCCTGCTGCCATGTCTGTGAGGGATTGTATTGACAGACAAGTAGTGTGT

AAGAGCCCACCCAGCCCACTGTGGACAGTGCTGTCACTGGCAGGTGGCCCTGGGTTATAAGAAAGCTGGCTTAGCATGAG

CCAGAGAAAGCAACCCAGTAAAAAGCATCCCTCCATGGTCTCTGCTTCAGCTCCTGACTCCTGGTCCCTTCGTTGGCTTC

TCTCAATAATGGACGATAATCTGTAAGCGGAATAAAACTTTTCATCCCCaagtggctttcggtcatgatgtttatcacag

caacagaaagcaaactaggacagtgtgcta

Aberrant plasticity of peripheral sensory axons in a painful neuropathy

Takashi Hirai, Yatendra Mulpuri, Yanbing Cheng, Zheng Xia, Wei Li, Supanigar Ruangsri, Igor Spigelman and Ichiro Nishimura

Fig. S3: The effect of alternative 3'UTR in chronic injury-related phenotypes. (**a**) DaPars identified mRNAs with significantly modified 3'UTR structures ($p<0.05$). Those mRNAs were predominantly processed with the alternative short 3'UTRs in the control axons. By contrast, the SNE-conditioned ipsilateral axons increased the use of alternative long 3'UTRs. (**b**) mRNA abundances in the axons with significant use of alternative 3'UTRs were cross-referenced for their steady state level. Only 5% and 10% of mRNAs with alternative 3'UTRs had their steady state levels changed in contra and ipsi axons, respectively. For those mRNAs with altered steady state levels, alternative long 3'UTRs were more frequently found upregulated, whereas those with alternative short 3'UTRs were relatively unchanged.

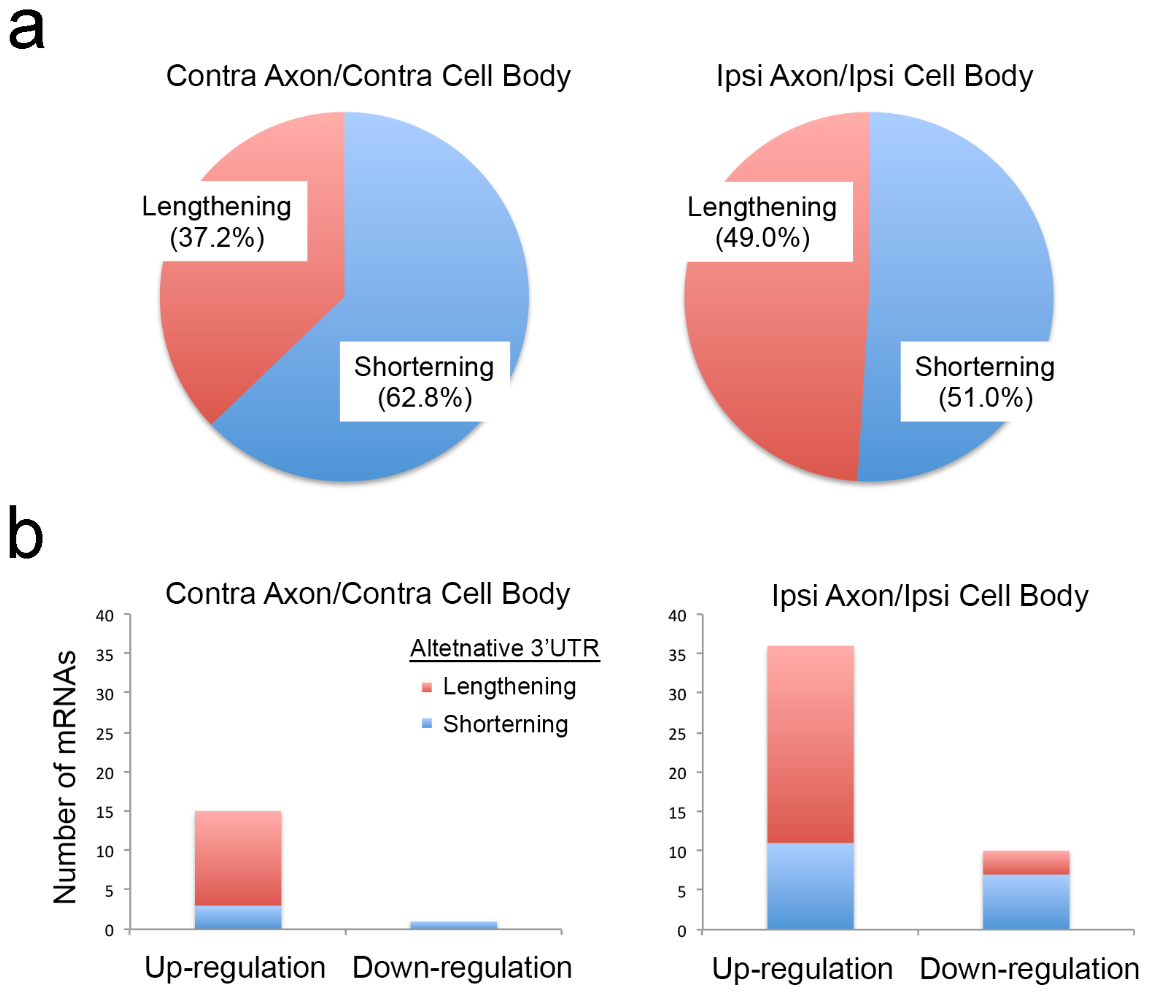

Aberrant plasticity of peripheral sensory axons in a painful neuropathy

Takashi Hirai, Yatendra Mulpuri, Yanbing Cheng, Zheng Xia, Wei Li, Supanigar Ruangsri, Igor Spigelman and Ichiro Nishimura

Fig. S4: Distribution of cis-elements for RNA binding proteins, ELAVL1, FUS, PTBP1 and Pum2 in the alternative 3'UTRs of rat NaV1.8.

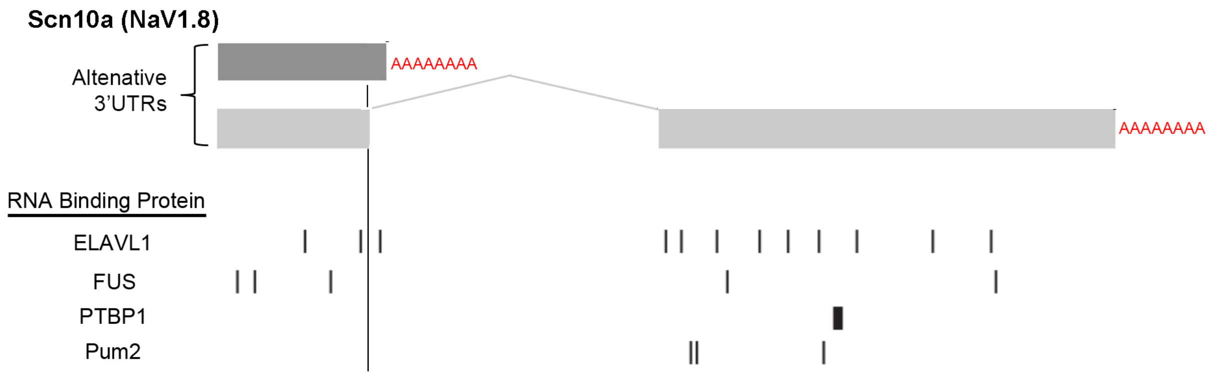

Fig. S5: The steady state mRNA levels of RNA binding proteins by RNA-seq data analysis.

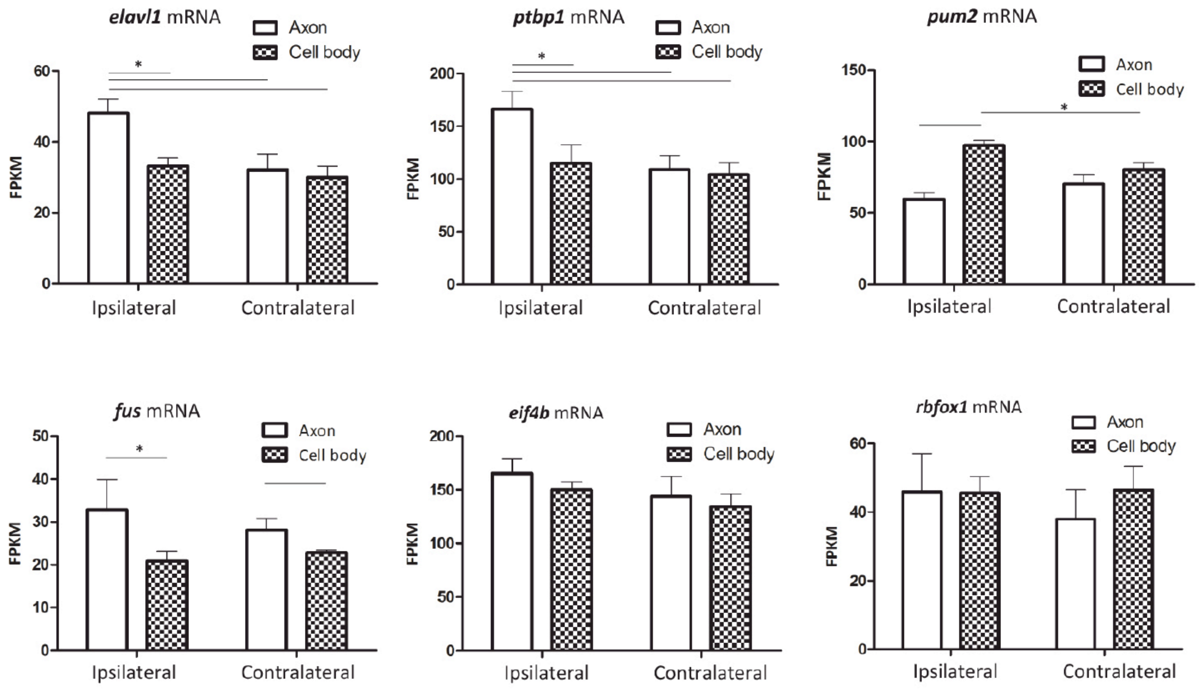

Supplement: Supplementary file 1 — Supplemental Information [file 41598_2017_3390_MOESM1_ESM.pdf]
